# Subject-specific variability in cerebrospinal fluid flow characteristics through cerebral aqueducts in a healthy population: a magnetic resonance imaging and computational investigation

**DOI:** 10.1007/s11517-025-03394-y

**Published:** 2025-06-12

**Authors:** Shusaku Maeda, Tomohiro Otani, Shigeki Yamada, Yoshiyuki Watanabe, Shigeo Wada

**Affiliations:** 1 Department of Mechanical Science and Bioengineering, Graduate School of Engineering Science, The University of Osaka, 1-3, Machikaneyama-Cho, Toyonaka, Osaka 560-8531 Japan; 2 Department of Neurosurgery, Graduate School of Medical Science, Nagoya City University, 1 Kawasumi, Mizuho-Cho, Mizuho-Ku, Nagoya, Aichi 467-8601 Japan; 3 Interfaculty Initiative in Information Studies/Institute of Industrial Science, The University of Tokyo, Tokyo, 112-0015 Japan; 4 Department of Radiology, Shiga University of Medical Science, Setatsukinowa-Cho, Otsu, Shiga 520-2192 Japan

**Keywords:** Cerebrospinal fluid, Stroke volume, Magnetic resonance imaging, Idiopathic normal pressure hydrocephalus, Computational fluid dynamics

## Abstract

Ventricular cerebrospinal fluid (CSF) flow has bi-directional flow profiles synchronized with cardiac pulsation. The increase in CSF stroke volume through the aqueduct (flow pathway between the third and fourth ventricle) is believed to be a biomarker of idiopathic normal pressure hydrocephalus (iNPH). However, several studies have reported that CSF stroke volume varies considerably even in healthy populations. To explore these variations from a fluid mechanics perspective, this study analyzed CSF flow characteristics in healthy individuals using magnetic resonance imaging (MRI)-based computational simulations. MRI data from 47 healthy subjects were acquired, and the maximum Reynolds number of the CSF flow through the aqueduct and the degree of CSF mixing were evaluated. Results showed that the Reynolds number of the CSF flow through the aqueduct was 28.6 ± 13.3, and the limited variation suggested fluid mechanical similarities of CSF flow characteristics in the healthy population. A positive correlation between CSF flow mixing and the Reynolds number was observed in both healthy populations and iNPH patients. These findings demonstrate that CSF flow through the aqueduct, especially in healthy populations, can be well characterized by examining fluid mechanical similarities.

**Graphical Abstract:**

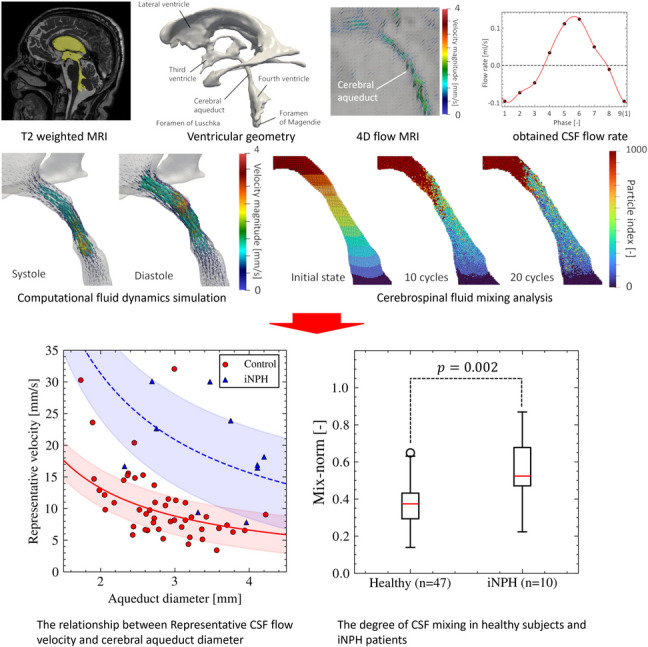

## Introduction

Intracranial cerebrospinal fluid (CSF) is a colorless fluid that fills the intracranial spaces and functions to transport nutrients and signaling molecules [1] and remove waste products [2]. CSF flow has a bidirectional profile that is synchronized with cardiac pulsation, and these flow characteristics in the ventricles, particularly through the aqueduct (the flow pathway between the third and fourth ventricles), can be observed using magnetic resonance imaging (MRI) [3]. A significant increase in CSF stroke volume through the aqueduct is a common biomarker of idiopathic normal pressure hydrocephalus (iNPH) [3, 4], also known as hyperdynamic flow. iNPH is a major form of NPH observed in older adult patients without a prior history of intracranial diseases, in which the abnormal accumulation of CSF in ventricles occurs within normal intracranial pressure ranges. From diagnostic and causal mechanistic perspectives, hyperdynamic CSF flow in iNPH patients and related physiological effects have attracted clinical [5–8] and biomechanical attention [9, 10].

CSF stroke volume in the healthy population is considered sufficiently low and is used as a control in comparisons with iNPH patients; yet, several MRI studies have reported considerable variation in healthy populations [7, 11, 12] depending on various factors, such as age [13–16] and sex [11]. The extent of variation in CSF stroke volume and correlations with various factors are inconsistent, even in recent large cohort studies [7, 17]; moreover, the physiological implications of this variation remain unclear. Because CSF stroke volume is an essential biomarker of iNPH, the ranges of these variations in healthy populations should be ascertained to enable quantitative evaluations for iNPH diagnosis. Because CSF flow characteristics are determined by not only flow volume rate but also body size from a fluid mechanistic point of view, we hypothesized that CSF flow characteristics in healthy populations would be clarified in terms of fluid mechanical similarities, despite the non-negligible variation in stroke volume.

Therefore, we aimed to examine subject-specific variability and similarities of CSF flow through the aqueduct in a healthy population using MRI and an MRI-based computational simulation. First, CSF flow variability acquired from 47 healthy subjects using MRI was summarized using not only stroke volume but also the maximum Reynolds number in a cardiac cycle to determine flow similarities. Subsequently, we conducted MRI-based computational simulations of CSF flow and evaluated the variability of CSF flow mixing in the aqueduct during multiple cardiac cycles and its association with the Reynolds number. These values and variabilities of the healthy population were compared with those of iNPH patients analyzed in a previous study [10].

## Methods

### Study participants

We included 19 healthy men (aged 24–92 years) and 28 women (aged 24–74 years) with no history of brain disorders or abnormal findings on MRI scans acquired at Shiga University of Medical Science Hospital. The study was approved by the Institutional Review Boards of Shiga University of Medical Science (No. R2019-227) and the University of Osaka (No. R2-1). Participants provided both verbal and written informed consent. This study was conducted according to the approved guidelines of the Declaration of Helsinki.

### MRI image analyses

#### Image acquisition

Images of each subject were acquired during normal sinus rhythm on a 3-T MRI scanner (Discovery MR 750 W, GE Healthcare, Milwaukee, WI, USA) with a 24-channel head coil. For the four-dimensional (4D) flow MRI, imaging data were obtained with 5 cm/s velocity encoding and synchronization with the peripheral pulse rate measured in the finger (repetition time [TR], 20.6 ms; echo time [TE], 2 ms; flip angle, 8°; field of view, 200 mm; matrix, 256 × 256; and voxel dimensions, 0.7813 × 0.7813 × 1.0 mm). The mean acquisition time for this sequence was approximately 8 min (range: 5–9 min) depending on heart rate. The field of view included the bilateral foramina of Monro and the upper cervical subarachnoid spaces in the sagittal plane, from which eight phases of the cardiac cycle were reconstructed. Aliasing artifacts and eddy-current-based phase offsets were eliminated using previously developed in-house software [18]. Additionally, to obtain volumetric data of the ventricular region, we acquired T2-weighted MRI data using a three-dimensional fast spin-echo sequence (TR, 2000 ms; TE, 86.7 ms; echo-train length, 100; field of view, 240 mm; matrix 288 × 288; voxel dimensions, 0.8 × 0.8 × 0.8 mm; and 228 slices). To check the consistency of the CSF flow velocity fields obtained from 4D flow MRI, we computed the CSF flow rate for four cross-sections of the aqueduct in each subject. Cases with a maximum error of > 25% during a cardiac cycle were excluded from subsequent analyses. Table 1 shows the physiological parameters and volumetric data obtained from the T2-weighted MRI images of the healthy participants included in the current study (*n* = 47) and those of the iNPH patients (*n* = 10) obtained previously [10] for comparison.

**Table 1:** Physiological parameters of the healthy participants (*n* = 47) and iNPH patients [10]

	Healthy subjects (*n* = 47)	iNPH [10] (*n* = 10)	*p*-value
Age	52 ± 18	75 ± 7	*p* < 1.0 × 10^–4^
Sex (male/female)	19/28	6/4	-
Weight [kg]	60 ± 11	56 ± 12	*p* = 0.674
LV volume [ml]	23.3 ± 11.8	109.7 ± 32.2	*p* < 1.0 × 10^–6^
3V volume [ml]	1.1 ± 0.5	4.5 ± 1.0	*p* < 1.2 × 10^–6^
4V volume [ml]	1.6 ± 0.3	3.0 ± 1.0	*p* < 3.6 × 10^–6^

Groups compared using two-tailed Mann–Whitney U tests

*LV* lateral ventricle, *3V* third ventricle, *4V* fourth ventricle

#### Image analysis

The MRI images were evaluated to determine the geometric characteristics of the aqueduct and the CSF flow characteristics. The average diameter of the aqueduct *D* was computed from T2-weighted MRI images, and CSF stroke volume and areal-averaged CSF flow velocity normalized to the cross-sectional area *U* were obtained from 4D flow MRI data. The CSF flow property through the aqueduct was characterized by the Reynolds number (Re), given by

1 $$\text{Re} = \frac{\rho UD}{\mu}$$

where *ρ* and *μ *are CSF density and viscosity, respectively. Based on experimental data, we modeled the CSF as a Newtonian fluid and defined *ρ* = 1 × 10^3^ kg/m^3^ and *μ* = 1 × 10^−3^ Pa·s [19]. Thus, the Reynolds number of CSF flow in the aqueduct can be understood as the relationship between aqueduct diameter *D* and flow velocity *U*.

### Computational simulation

#### Computational framework

**Fig. 1:**
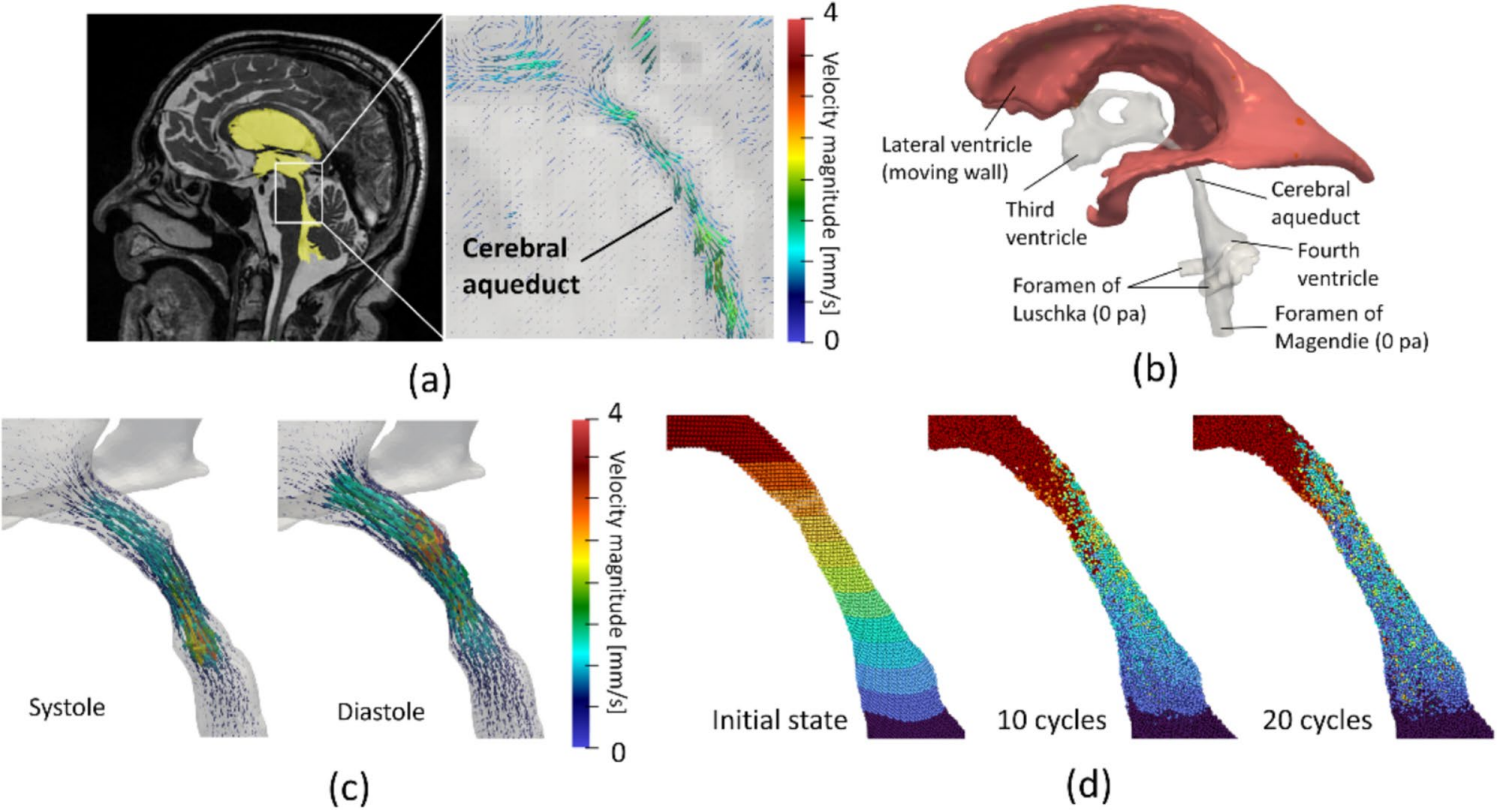
Computational framework for analysis of CSF mixing in the cerebral aqueduct. **a** The ventricular geometry was extracted from T2-weighted MRI images, and the CSF flow profile through the aqueduct was measured using 4D flow MRI. **b** Computational fluid dynamics simulations of ventricular CSF flow were performed, setting the moving wall boundary condition at the lateral ventricular wall (red region) and the Dirichlet pressure boundary condition of 0 Pa at the Luska and Magendie foramen cross sections. **c** We obtained the CSF flow velocity field during one cardiac cycle. **d** Massless particle tracking was performed during 20 cardiac cycles, with the particles placed uniformly in the ventricular region at the initial phase

A subject-specific computational simulation of the ventricular CSF flow was conducted to analyze CSF flow mixing states in the aqueduct using a previously developed framework [10]. This is summarized in Fig. 1. Subject-specific geometries of the brain ventricles from the lateral ventricle to the foramen of Magendie and Luschka were extracted using T2-weighted MRI (Fig. 1a) and reconstructed as a set of triangular surfaces using Mimics (version 23; Materialize Inc., Yokohama, Japan; Fig. 1b). The CSF domains were spatially discretized using first-order tetrahedral elements with a base mesh size of 0.7 mm and three layers of boundary prism layer elements generated with Hypermesh (version 2022; Altair Inc., Troy, MI, USA), based on a mesh size independency test performed in our previous study [10]. In this test, we compared three cases of computational results of the ventricular CSF flow using coarse, medium, and fine meshes (i.e., base mesh sizes of 1.4 mm, 0.7 mm, and 0.35 mm, respectively), and confirmed that the error between the cases with fine and medium mesh was within 5%. The CSF was modeled as incompressible Newtonian fluids, and this flow was expressed by the equation of continuity and incompressible Navier–Stokes equation in the arbitrary Lagrangian–Eulerian (ALE) form, given by:

2 $$\nabla \cdot {\mathbf{v}} = 0,$$

3 $$\rho \left( {{\partial_t}{\mathbf{v}} + {\mathbf{\tilde v}} \cdot \nabla {\mathbf{v}}} \right) = - \nabla p + \mu {\nabla^2}{\mathbf{v}},$$

where $\mathbf{v}$ is the velocity vector, $\mathbf{\tilde v}$ is the advection velocity in ALE coordinates, and $p$ is the pressure. These equations were spatially discretized using the finite element method with a streamline upwind Petrov–Galerkin formulation [20] and solved using a fractional step method in time. The advection term was treated explicitly, whereas the diffusion term was handled using the Crank–Nicolson method. For computational details, please see our previous study [10]. For the boundary conditions, a moving wall boundary condition was imposed in the lateral ventricles to account for transient volume changes through the aqueduct measured using 4D flow MRI (Fig. 1a, b). The no-slip boundary condition was applied to the entire ventricle wall, and a constant 0 Pa pressure was applied to the cross-sectional plane in the foramen of Magendie and Luschka (Fig. 1b). From the obtained CSF flow velocity fields (Fig. 1c), CSF mixing state was evaluated using massless particle tracking (Fig. 1d). At the initial phase, the tracers were uniformly placed at 0.25 mm intervals along each of the three Cartesian axes throughout the ventricles, and these trajectories were computed by the CSF flow velocity fields.

#### Mixing analysis

To quantitatively evaluate CSF flow mixing in the aqueduct during multiple cardiac cycles, we computed the particle mixing in the evaluation domains and quantified these using the mix-norm [21, 22], which is an index of multiscale flow mixing that ranges from 0 (completely separated) to 1 (completely mixed) that is intuitively understood as the particle exchange ratio in the target domain from initial states.

**Fig. 2:**
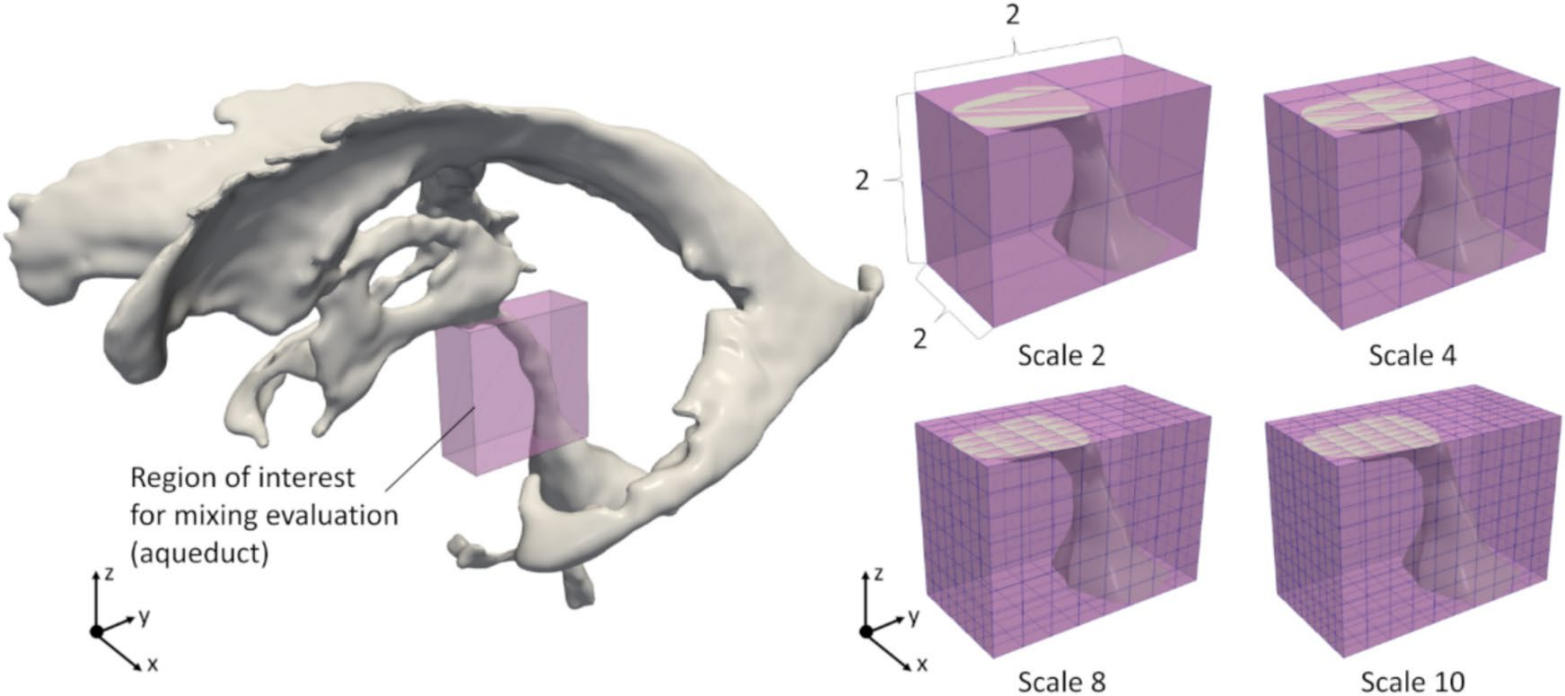
Computational domain for calculating mix-norm. The number of scales represents the division number along each Cartesian coordinate direction for the bounding box of the cerebral aqueduct (i.e., the evaluation region)

The computational procedures of the mix-norm were as follows. In preparation, the evaluation domain, including the aqueduct, was assigned as Cartesian domain $\Gamma$ and divided into equally spaced subdomains $\Gamma_{i,j}$ (Fig. 2), where *i* (= 2, 4, 6, 8, and 10) represents the number of divisions along each edge of $\Gamma$, and *j* is the index of each subdomain ($j \in \left[ {1,i \times i \times i} \right]$). The degree of particle mixing *c* within each subdomain $\Gamma_{i,j}$ from the initial state to time *T* was defined as the percentage of particles that remain in $\Gamma_{i,j}$ at time *T* relative to the initial state, such that

4 $$c\left( T \right) = \frac{{{N_{{\text{out}}}}}}{{{N_{{\text{in}}}} + {N_{{\text{out}}}}}}$$

where ${N_\text{in}}$ and ${N_\text{out}}$ are the numbers of particles that were initially inside and outside of $\Gamma_{i,j}$, respectively. The mixing degree at each scale *i*, *m*_*i*_, was computed as the root mean square of $c$ over all subdomains at that scale:

5 $${m_i} = {\left( {\sum\limits_{j = 1}^{i \times i \times i} {{c_j}^2} } \right)^\frac{1}{2}}$$

Finally, the original mix-norm *m* was defined as the root mean square of *m*_*i*_ over all scales:

6 $$m = {\left( {\sum\limits_{i = 1}^5 {{m_{2i}}} } \right)^\frac{1}{2}}$$

In this study, we evaluated not only the original mix-norm *m* but also *m*_*i*_ to investigate the mixing degree at each scale.

### Statistical analysis

We examined sex and age differences in healthy subjects, as well as differences between healthy subjects and iNPH patients. For the effect of age, healthy subjects were classified into young (< 65 years old) and older adult (≥ 65 years old) groups. Differences between groups were analyzed using two-tailed Mann–Whitney U tests, with a critical *p*-value of 0.05. Subjects that fell outside of the interquartile range were considered outliers. The relationship between *U* and *D* was evaluated using linear regression because the Reynolds number was determined by this relationship (Eq. 1). The linear regression analysis was performed using Scikit-learn (version 0.24.2) [23], and the Mann–Whitney U tests were performed using SciPy (version 1.7.3) [24]. All parameters are reported as means and standard deviations unless otherwise noted.

## Results

**Fig. 3:**
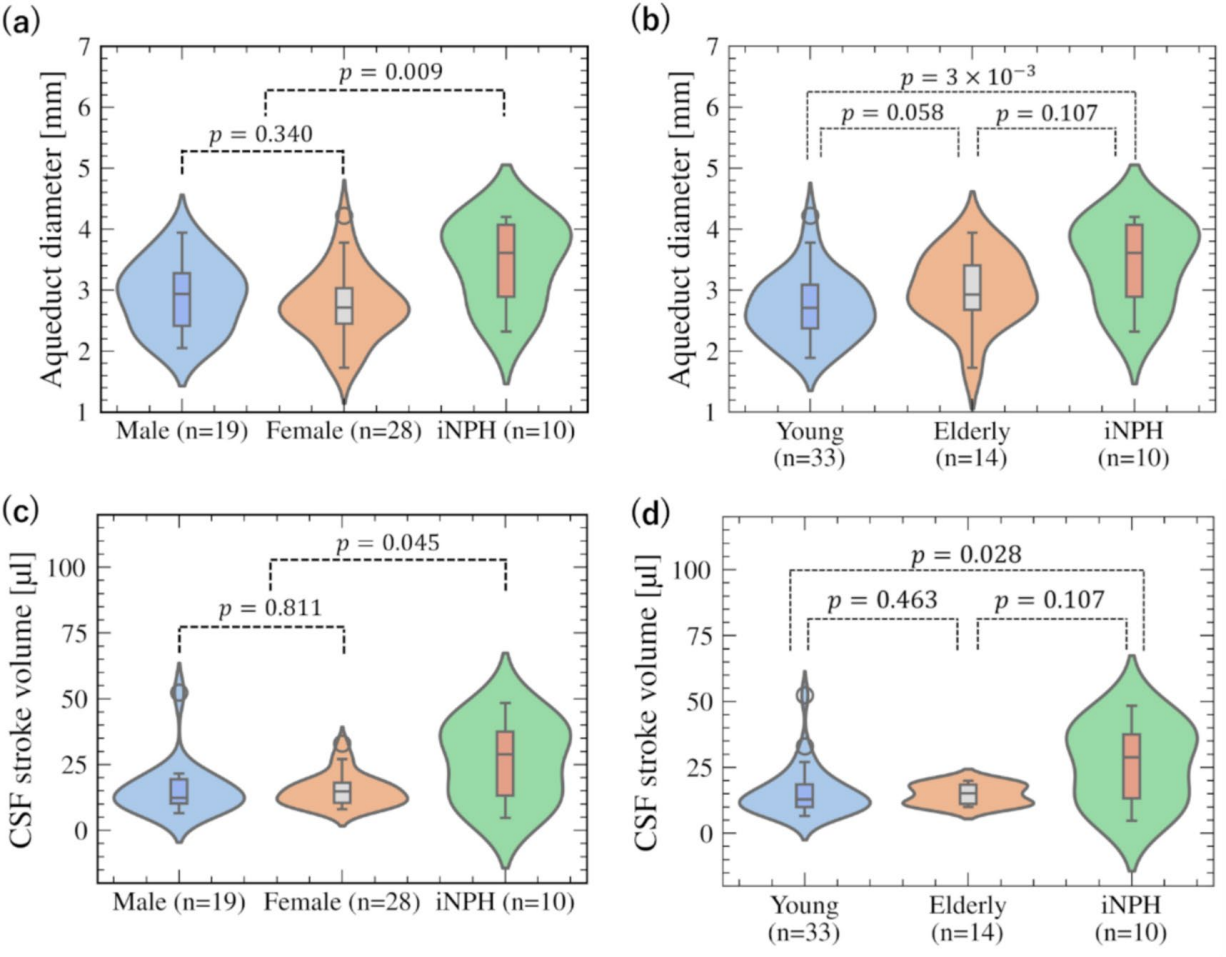
Distributions of aqueduct diameter and CSF stroke volume in healthy and iNPH groups. **a** Violin plots of aqueduct diameter for the male, female, and iNPH groups. **b** Violin plots of aqueduct diameter for the young adult, older adult, and iNPH groups. **c** Violin plots of CSF stroke volume for the male, female, and iNPH groups. **d** Violin plots of CSF stroke volume for the young adult, older adult, and iNPH groups

Figure 3 summarizes the aqueduct diameter *D* and CSF stroke volume by sex and age. Values of the iNPH patients reported previously [10] have also been provided for comparison. For geometric variability, *D* was 2.82 ± 0.56 mm (2.90 ± 0.54 mm for men, 2.78 ± 0.56 mm for women, 2.72 ± 0.54 mm for the young group, and 3.03 ± 0.57 mm for the older adult group). There were no significant differences between the two sexes (*p* = 0.34; Fig. 3a) or age groups (*p* = 0.058; Fig. 3b). For CSF flow characteristics, the CSF stroke volume was 15.3 ± 7.8 µl (15.5 ± 9.8 µl for men, 15.2 ± 6.0 µl for women, 15.5 ± 9.1 µl for the young group, and 14.7 ± 3.8 µl for the older adult group). There were no significant differences between the two sexes (*p* = 0.811; Fig. 3c) or age groups (*p* = 0.463, Fig. 3d).

**Fig. 4:**
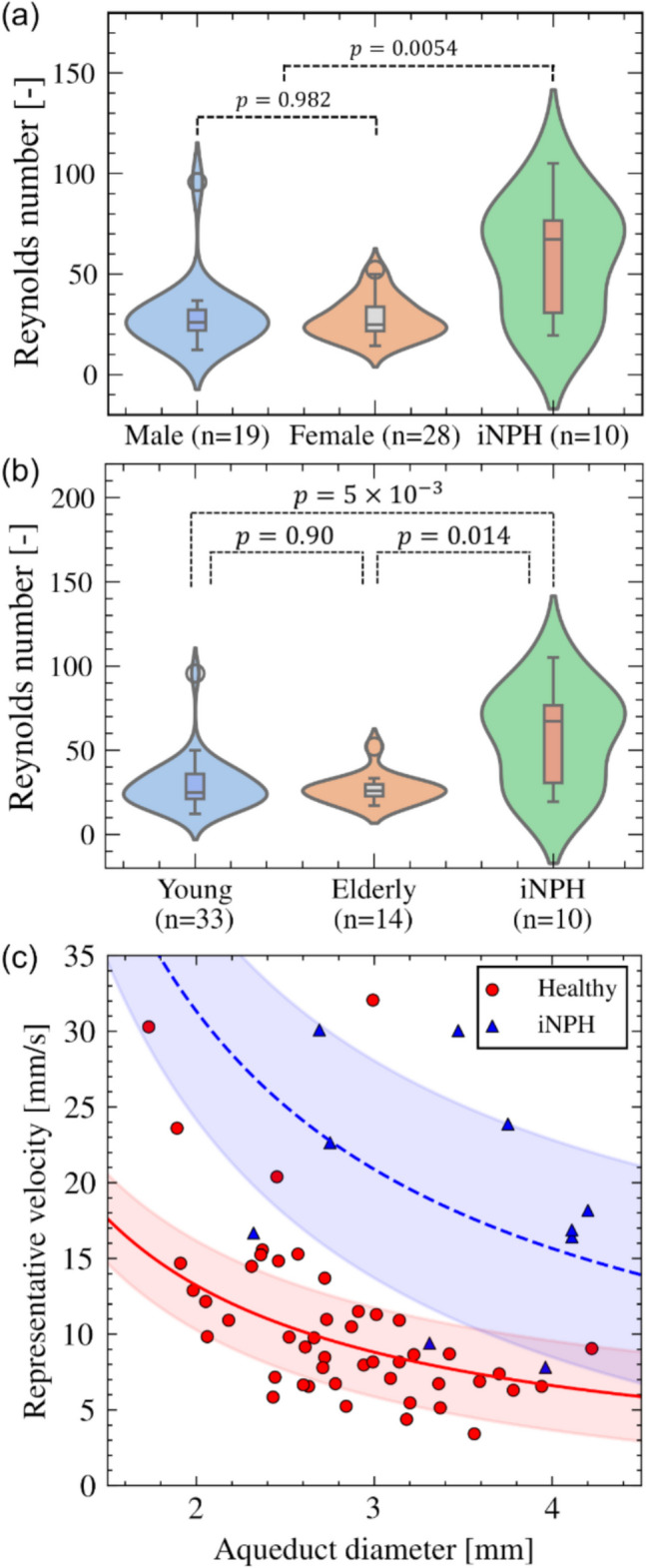
Distributions of Reynolds number and the relationship between aqueduct diameter and representative CSF velocity in healthy and iNPH groups. **a** Violin plots of the Reynolds number for the male, female, and iNPH groups. **b** Violin plots of the Reynolds number for young adult, older adult, and iNPH groups. **c** Scatter plots showing the relationship between aqueduct diameter and representative CSF velocity

To characterize the CSF flow dynamics based on the above parameters, we computed the maximum Reynolds number in a cardiac cycle for each subject (Fig. 4). The average maximum Reynolds number was 28.6 ± 13.3 (29.4 ± 17.1 for men, 28.0 ± 10.0 for women, 29.1 ± 15.1 for the young group, and 27.0 ± 8.90 for the older adult group). There was no significant difference between the sexes (*p* = 0.982; Fig. 4a) or age groups (*p* = 0.90; Fig. 4b). Figure 4c shows a scatterplot of the relationship between aqueduct diameter and representative CSF velocity with the regression curves (*U* = *a*/*D*, where *a* is a coefficient). The resulting constant *a* was 26.4 (R^2^ = 0.324; 29.5 for men [R^2^ = 0.448] and 27.8 for women [R^2^ = 0.350]). The Reynolds number of iNPH patients was 58.0 ± 27.6 and significantly higher than that of healthy subjects. However, there was no significant correlation between *U* and *D* (a = 62.6, R^2^ = − 3.5 × 10^−3^).

**Fig. 5:**
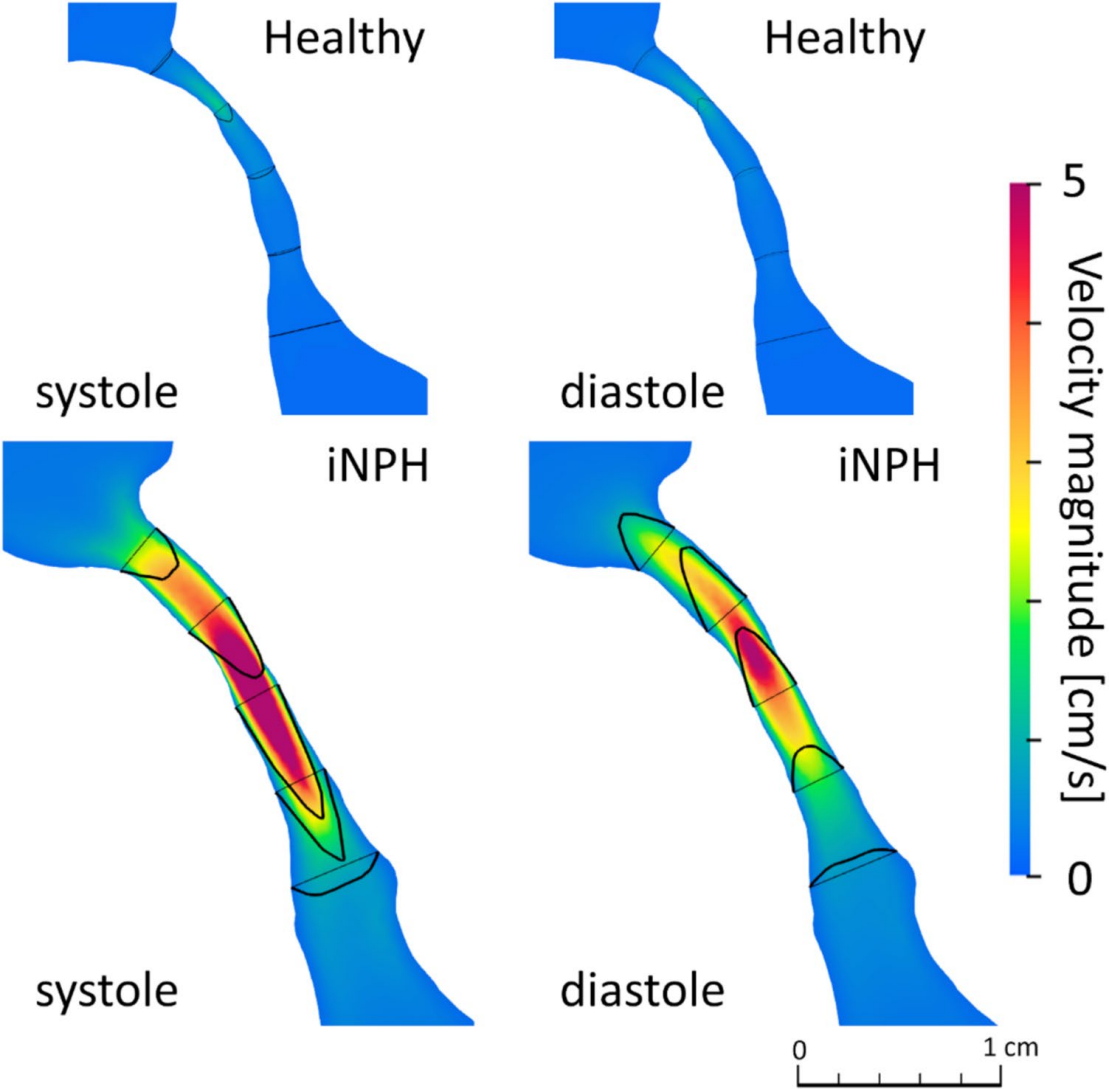
Representative snapshots of the flow velocity profile on the mid-sagittal plane of the aqueduct in a healthy subject (top) and an iNPH patient (bottom) at systolic and diastolic phases

Representative snapshots of CSF flow velocity profiles through the aqueduct in the mid-sagittal plane in a healthy subject and an iNPH patient, computed in [10], are shown in Fig. 5 for comparison. In healthy subjects, the CSF flow velocity showed symmetrical parabolic profiles in both systolic and diastolic phases, with the highest magnitude around the center of the aqueduct. In contrast, that of iNPH patients was relatively high, and the flow velocity profiles became asymmetric in the aqueduct cross-sections because of inertial effects.

**Fig. 6:**
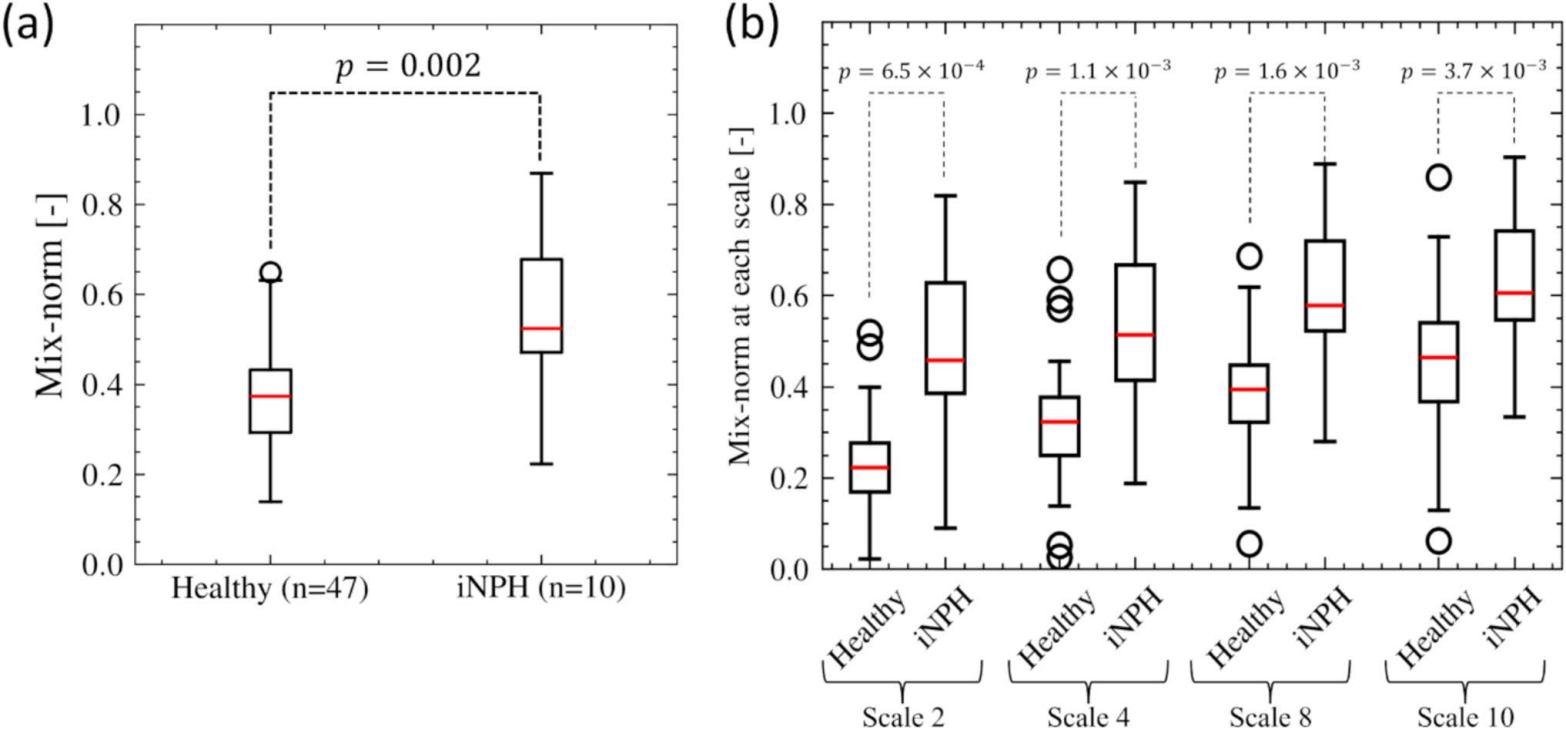
Distributions of the index for the degree of CSF mixing in healthy and iNPH groups. **a** Boxplots of mix-norm for healthy subjects and iNPH patients. **b** Boxplots of mix-norm for each scale (2, 4, 8, and 10) for healthy subjects and iNPH patients

Figure 6 shows the mix-norm and mixing degrees for each scale (*i* = 2, 4, 8, and 10) in the healthy subjects and iNPH patients [10] for comparison. The mix-norms differed significantly between the healthy subjects (0.361 ± 0.101) and iNPH patients (0.554 ± 0.183; Fig. 6a). For the mixing degrees of each scale (Fig. 6b), there was no significant difference between healthy men and women but were significantly lower in the healthy subjects than in iNPH patients, especially for the large scale at *i* = 2 (0.226 ± 0.098 in healthy subjects and 0.474 ± 0.203 in iNPH patients, *p* = 6.5 × 10^−4^).

**Fig. 7:**
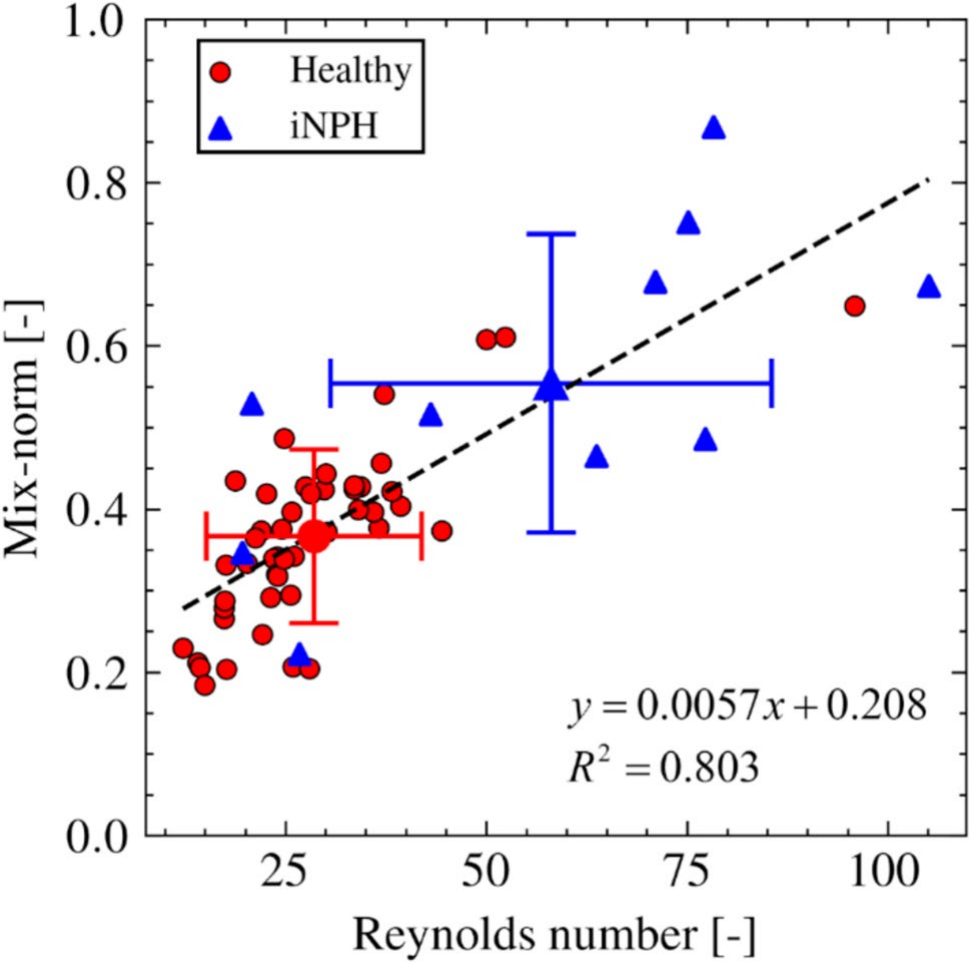
Scatter plot of the relationship between Reynolds number and mix-norm. Error bars represent the standard deviations of each variable, and the intersection points correspond to the mean values

Finally, Fig. 7 summarizes the relationship between the mix-norm and Reynolds number in healthy subjects and iNPH patients. Positive correlations were observed in the healthy and iNPH groups (y = 0.006x ± 0.208, R^2^ = 0.803 for all subjects). There was a wider range of standard deviations in iNPH patients than in healthy subjects for the Reynolds number and mix-norm.

## Discussion

This study aimed to clarify the extent of variation in CSF flow characteristics in the aqueduct in a healthy population. Our results showed that CSF stroke volume in the healthy population exhibited a five-fold larger variation between the maximum and minimum values than patients, with only a small or no significant dependency on age and sex (Fig. 3). These properties are consistent with previous observations of CSF flow dynamics in healthy subjects [7, 12, 17]. However, a significant inverse relationship was found between *D* and *U* in healthy subjects, with a resulting Reynolds number of 28.6 ± 13.3 (Fig. 4). This inverse relationship indicated that the CSF flow profiles of healthy subjects have fluid mechanical similarities characterized by the Reynolds number despite the non-negligible variation in both *D* and *U*.

To consider the implication of the above similarities in healthy populations, we evaluated similarities in iNPH patients based on our previous study [10]. In contrast to the healthy population, iNPH patients showed a relatively high Reynolds number with large variation (58.0 ± 27.5), and there was no significant inverse relationship between *D* and *U* (Fig. 4). These results suggest that the CSF flow characteristics of iNPH patients had high subject-specific variability and that pathological changes in the CSF flow profiles of iNPH patients can be understood as a deviation from normal ranges of fluid mechanical similarity observed in healthy populations.

The concept of fluid mechanical similarity can characterize CSF flow profiles and may thus have physiological implications. Because intracranial CSF functions to signal transport and remove waste products, the CSF components in each ventricle and the extent of these exchanges have attracted considerable attention [25–27]. We previously reported the presence of abnormal CSF mixing through the aqueduct in iNPH patients by introducing the mix-norm [10]; however, the mechanical implications were not clarified owing to the lack of healthy population data. Our current study addressed this limitation and obtained results that showed a strong positive correlation between the Reynolds number and the mix-norm (Fig. 7). This finding is mechanically reasonable because flow instability increases with increasing Reynolds number, as observed in iNPH patients (Fig. 5), and the increase in the mix-norm can be understood as a change in particle trajectories from steady closed to open profiles. Our finding suggests that the extent of CSF flow mixing through the aqueduct can be characterized by fluid mechanical similarities using the Reynolds number.

This study has four main limitations. First, several healthy subjects had a relatively high stroke volume that was comparable to that of the iNPH patients; moreover, their Reynolds numbers were considered outliers (Fig. 4). Most of our healthy subjects had smaller Reynolds numbers than iNPH patients, which is consistent with previous reports [3, 4]. Therefore, the outlier subjects may have fallen outside of the normal range. Further follow-up studies are required to clarify whether such abnormal flow patterns are merely a subject-specific property in the healthy range or early signs of a neurological disorder. Second, this study was limited to the aqueduct, and whole ventricular CSF flow profiles were outside of the scope of the current study. Although ventricular CSF flow is relatively slow, except in the aqueduct, further consideration of the CSF flow of the entire ventricular system on a longer time scale would provide valuable insight into the mass and signal transport functions of the CSF. Third, although the main aim of this study was to investigate variability in healthy subjects, comparison with a larger dataset of iNPH patients would be valuable. Furthermore, because alterations in CSF flow properties may be related to pathological progression, iNPH patients should be classified according to disease grade. Fourth, our simulation represented pulsatile CSF flow by changing the lateral ventricle volume and treating other wall boundaries as rigid. Ventricular wall motion results from brain tissue deformation due to cardiac pulsation [28] and thus fluid–structure interactions of brain tissue, blood, and CSF need to be considered to determine physiologically accurate CSF flow mechanics, especially in the entire intracranial region.

## Conclusions

In the current study, we examined the aqueductal CSF flow characteristics of healthy subjects to determine the effects of subject-specific fluid dynamics variations. To evaluate CSF flow characteristics, excluding the effect of body size, we used the Reynolds number, which is a non-dimensional index based on the product of aqueduct diameter and CSF flow velocity. We found that although CSF stroke volume and aqueduct diameter had non-negligible variations, these values were inversely correlated, and the variation in the Reynolds number was relatively limited. The above inverse relationship was not observed in iNPH patients. These results suggest that subject-specific variation in CSF stroke volume is influenced by body size and that CSF flow characteristics, such as CSF mixing degree, have fluid dynamical similarities across healthy subjects.

## Data Availability

The data that supports the findings of this study are available on request from the corresponding author upon reasonable request. The data are not publicly available because of ethical restrictions.
